# Bone morphogenetic protein 4 rescues the bone regenerative potential of old muscle-derived stem cells via regulation of cell cycle inhibitors

**DOI:** 10.1186/s13287-022-03047-z

**Published:** 2022-07-30

**Authors:** Haizi Cheng, Xueqin Gao, Matthieu Huard, Aiping Lu, Joseph J. Ruzbarsky, Sara Amra, Bing Wang, Johnny Huard

**Affiliations:** 1 Department of Orthopaedic Surgery, McGovern Medical School, The University of Texas Health Science Center at Houston, Houston, TX USA; 2 School of Pharmacy, University of Pittsburgh, Pittsburgh, PA USA; 3 Linda and Mitch Hart Center for Regenerative and Personalized Medicine, Steadman Philippon Research Institute, 181 W Meadow Dr, Suite 1000, Vail, CO 81657 USA; 4 The Steadman Clinic, Aspen, CO USA; 5 AAV Vector Gene Core, Department of Medicine, University of Pittsburgh, Pittsburgh, PA USA

**Keywords:** Bone morphogenetic protein 4, Bone regeneration, P16, P18, Aging, Muscle-derived stem cells

## Abstract

**Background:**

Bone morphogenetic protein 4 (BMP4) promotes the osteogenic differentiation and the bone regenerative potential of muscle-derived stem cells (MDSCs). BMP4 also promotes the self-renewal of both embryonic and somatic stem cells; however, BMP4 signaling activity significantly decreases with age. Cyclin-dependent kinase inhibitors P16^INK4A^ (P16) and P18^INK4C^ (P18) induce early G1-phase cell cycle blockade by targeting cyclin-dependent kinase 4/6. It is still unclear if BMP4 affects the bone regenerative potential of old MDSCs through regulation of P16 and P18 expression.

**Methods:**

Young and old MDSCs were isolated from 3 week (young) and 2-year-old (old) mice. In vitro cell proliferation and multipotent differentiation were performed for young and old MDSCs both before and after BMP4/GFP transduction. Cell cycle genes were analyzed using Q-PCR. The bone regenerative potential of young and old MDSCs transduced with BMP4/GFP were compared using Micro-CT and histological analysis. The bone regenerative potential of young and old MDSCs was also compared between single and double transduction (higher BMP4 levels expression). The cell proliferation, mitochondrial function and osteogenic differentiation was also compared in vitro between cells that have been transduced with BMP4GFP (single and double transduction). The correlation of bone regeneration capacity of young and old MDSCs with P16 and P18 expression was further evaluated at 10 days after cell transplantation using histology and western blot analysis.

**Results:**

Old murine MDSCs (MDSCs) exhibit reduced proliferation and multi-lineage differentiation potential with or without BMP4 stimulation*,* when compared to young murine MDSCs. Old MDSCs express significantly higher P16 and lower P18, with more cells in the G0/1 phase and fewer cells in the G2/M phase, compared to young MDSCs. Old MDSCs retrovirally transduced to express BMP4 regenerated less bone in a critical size skull defect in CD-1 nude mice when compared to young retrovirally transduced MDSCs expressing similar BMP4 levels and contribute less to the new regenerated new bone. Importantly, both young and old MDSCs can regenerate more bone when BMP4 expression levels are increased by double-transduction with the retroviral-BMP4/GFP. However, the bone regeneration enhancement with elevated BMP4 was more profound in old MDSCs (400% at 2 weeks) compared to young MDSCs (200%). Accordingly, P18 is upregulated while P16 is downregulated after BMP4 transduction. Double transduction did not further increase cell proliferation nor mitochondrial function but did significantly increase Osx expression in both young and old MDSCs. Old MDSCs had even significant higher Osx levels as compared to young MDSCs following double transduction, while a similar Alp expression was observed between young and old MDSCs after double transduction. In addition, at 10 days after cell transplantation, old MDSCs having undergone double transduction regenerated bone more rapidly as showed by Alcian blue and Von Kossa staining. Western blot assays demonstrated that old MDSCs after retro-BMP4/GFP double transduction have significantly lower P18 expression levels when compared to young BMP4-transduced MDSCs. In addition, P18 expression was slightly increased in old MDSCs after double transduction when compared to single transduction. P16 expression was not detectable for both young and two old BMP4/GFP transduced MDSCs groups.

**Conclusions:**

In summary, BMP4 can offset the adverse effect of aging on the osteogenic differentiation and the bone regenerative potential of old MDSCs via up-regulation of P18 and down-regulation P16 expression.

## Introduction

Muscle-derived stem cells (MDSCs) have been isolated and investigated to improve the repair of a variety of tissues. Most MDSC-based gene therapy and tissue engineering applications have used MDSCs isolated from young animals [1–12]. However, MDSCs isolated from naturally aged and progeroid mice have reduced proliferative and myogenic differentiation capacities [13]. MDSCs isolated from progeroid mice display reduced muscle regenerative potential when compared to MDSCs isolated from age matched normal animals [13, 14].

Previous work has shown that murine or human MDSCs retrovirally transduced to express bone morphogenetic protein 4 (MDSCs/BMP4/GFP) could efficiently promote complete healing of critical sized 5-mm calvarial defects of SCID or C57BL/6 J mice at three or six weeks after injury [5, 15–25]. Old human MDSCs have also been shown to be as effective as young MDSCs in regenerating bone in both young and old mice when transduced with lenti-BMP2 [26].

BMPs have also been shown to promote the self-renewal of both embryonic and somatic stem cells [27–29]. Furthermore, increasing BMP signaling can even prolong the lifespan and proliferation of Drosophila ovarian germline stem cells (GSCs) [3]. However, BMP signaling activity significantly decreases with age. Additionally, cyclin-dependent kinase inhibitors P16^INK4A^ (P16) and p18^INK4C^ (P18) induce early G1-phase cell cycle blockade by targeting cyclin-dependent kinase (CDK) 4/6 [30–34]. Since BMPs can play a role in mitigating the effects of aging [26], it is hypothesized that BMP may rejuvenate old MDSCs by increasing their osteogenic potentials and bone regenerative capacities through modulation of P16 and P18.

## Materials and methods

### Isolation and culture of MDSCs

Since it has been previously shown that male MDSCs are more osteogenic than female MDSCs, only male MDSCs were used for the current study [5]. MDSCs were isolated from three 3-week-old (young) and three 2-year-old (old) C57BL/10 J male mice using a modified preplate technique [35]. Briefly, the skeletal muscle tissues were minced with scissors mechanically and digested enzymatically with collagenase XI, dispase, and trypsin–EDTA sequentially. The resultant muscle slurry was plated on collagen type I coated flasks. The non-adhered cells were replated at 2 h, and 1,2,3,4, and 5 days by spinning down and refreshing with new medium. The cells that eventually adhered after 5 days are defined as muscle-derived stem cells (MDSC, also called slow adhering cells, SAC). MDSCs were cultured on collagen-coated flasks in proliferation medium containing phenol red high-glucose Dulbecco’s Modified Eagle’s Medium (DMEM) (Invitrogen) supplemented with 20% fetal bovine serum (FBS) (Invitrogen), 1% penicillin/streptomycin (Invitrogen), and 1% chick embryo extract (Accurate Chemical Co.). Once cells reached 50% confluence, they were detached with 0.1% trypsin–EDTA (Invitrogen) and replated at a density of 200 cells/cm^2^ and cultured at 37 °C, 5% CO_2_ and passaged every day for expansion.

### Cell proliferation assay

MDSCs were seeded in a collagen type I coated 12-well plates at a density of 2000 cells per 24-plate well with 7 replicate wells. By using a live imaging system [36] (Automated Cell Technologies) equipped with a 20 × objective, images were acquired at 10 min intervals for 48 h. Seven images were taken automatically from each well at each time point and analyzed for both the young and old MDSC populations. For cell proliferation, the average number of cells from 7 replicate wells at each time point was calculated. Experiments were repeated three times. To estimate the population doubling time, experimental data sets for each population were used to curve-fit to the rearranged Sherley model [36, 37].

### Myogenic differentiation

MDSCs were seeded in a type I collagen coated 12-well-plate at a density of 5000 cells/well in proliferation medium (PM). Three replicate wells were set for each population of either young or old MDSCs. After the cells reached 100% confluence, the cells were switched to myogenic medium which contains high-glucose DMEM with 2% FBS and 1% penicillin/streptomycin (Invitrogen). The medium was changed three times per week for a total of 7 days. Cells were fixed with cold methanol for 2 min and blocked with 5% donkey serum. Cells were incubated with mouse anti-fast myosin heavy chain (fMHC) (M4276, 1:250; Sigma-Aldrich) for 1 h and subsequently incubated with goat anti-mouse biotinylated IgG (BA-1000, Vector Laboratories, 1:250 dilution) for 1 h and washed, followed by streptavidin-594 (1:500; Sigma-Aldrich) for 15 min to fluorescently label the fMHC. After another wash, cells were then incubated with 4′, 6′ diamidino-2-phenylindole (DAPI, 100 ng/ml; Sigma-Aldrich) for 10 min at room temperature to reveal nuclei. Mouse IgG isotype was used to replace the fMHC primary antibody as a negative control. Fluorescent images were captured and processed by utilizing a Nikon Eclipse Ti2 inverted microscope. Six images were taken at different area of each well. The experiments were repeated three times for both the young and old MDSCs populations. To quantify the myogenic differentiation, the percentage of fMHC expressing cells was calculated. Total cell number was determined by the number of the DAPI positive nuclei.

### Osteogenic differentiation of MDSCs in vitro

Cells were seeded in the type I collagen coated 6-well plates at a density of 10,000 cells/well. After the cells reached 100% confluence, the cells were switched to an osteogenic medium which contains 10 ng/ml BMP4, high-glucose DMEM with 10% FBS, 1% penicillin/streptomycin, 10^–2^ M β-glycerophosphate, 50 µg/ml ascorbic acid-2 phosphate, and 10^–7^ M dexamethasone (all from Sigma-Aldrich). The medium was changed three times a week for a total of 7 days. Cells were fixed with neutral buffered formalin (NBF). Osteogenic potential was determined using Sigma Alkaline Phosphatase (ALP) staining kit (86R-1kt) and purple pixel-positive area percentage was quantified for each young and old MDSCs population.

### Chondrogenic differentiation of MDSCs in vitro

Chondrogenic differentiation was performed according to previously described protocol [24]. Briefly, 1.25 × 10^5^ cells were aliquoted into 15-ml tubes with 4 pellets for both the young and old MDSC populations. After centrifugation at 800 g for 5 min, cells were resuspended in StemPro® chondrogenic medium and centrifuged at 500 g for 5 min. The medium was changed every 2–3 days and chondrogenesis evaluated at 28 days after chondrogenic differentiation. All the pellets were fixed in neutral buffered formalin at room temperature for 1 h, then embedded in NEG freezing medium and then snap-frozen in liquid nitrogen. Sections were cut in 10 μm thickness for Alcian blue staining (Alcian blue 8GX 1 g, Acetic acid 3%, pH 2.5). The percentage of Alcian blue-positive area of all pellets was quantified using Nikon NIS software.

### Adipogenic differentiation of MDSCs in vitro

Cells were seeded in the type I collagen coated 6-well plates at a density of 10,000 cells/well. After the cells reached 100% confluence, the cells were switched to StemPro® Adipogenesis Differentiation medium and medium was changed every 3–4 days. Oil Red staining was performed to detect the adipogenic differentiation at day 14. Adipogenesis was determined by Oil Red staining and quantified by calculating the percentage of Oil Red-stained area from 3 replicate wells of both young and old MDSCs.

### Flow cytometric analysis of cell cycle

MDSCs were harvested and washed in PBS. Cold 70% ethanol was used to fix and permeabilize the cells for 30 min at 4 °C. Cells were centrifuged at 850 g to remove the ethanol, and then washed twice in PBS. By using the Propidium Iodide (PI) Flow Cytometry Kit (ab139418, Abcam), cells were treated with ribonuclease to remove RNA. 200 µl PI was added to further stain the DNA for flow cytometry analysis.

### Quantitative real-time PCR (qPCR) analysis

RNA was extracted using TRIzol reagent (Invitrogen) following the manufacturer's protocol, cDNA synthesis was performed using the Iscript cDNA synthesis kit (Bio-Rad). Quantitative PCR was performed by using the SsoAdvanced™ Universal SYBR® Green Supermix (Bio-Rad). The relative quantification was normalized to glyceraldehyde-3-phosphate dehydrogenase (GAPDH). Primers were designed using Primer 3 [38, 39]. Primers sequences are shown in Table 1.

**Table 1:** Primers’ information

Gene ID	Accession #	Forward primer (5′–3′)	Reverse primer (5′–3′)
P18	BC027026	CTGGAGTTCCAGGCTGATGT	GCAGGCTGTGTGCTTCATAA
P16	BC058190	GTCGCAGGTTCTTGGTCACT	CGAATCTGCACCGTAGTTGA
Alp	XM124424	CCGATGGCACACCTGCTT	GAGGCATACGCCATCACATG
Col1	NC000077	GACGCCATCAAGGTCTACTG	ACGGGAATCCATCGGTCA
Osx	NM130458	CCCTTCTCAAGCACCAATGG	AGGGTGGGTAGTCATTTGCATAG
Runx2	NM_001146038.2	CCCAGCCACCTTTACCTACA	TATGGAGTGCTGCTGGTCTG
Ocn	NM_007541.3	CAGTATGGCTTGAAGACCGC	AGAGAGAGAGGACAGGGAGG
GAPDH	BC145812.1	CCGGGGCTGGCATTGCTCTC	GTGTTGGGGGCCGAGTTGGG

### Retroviral vector construction

We used a retroviral vector containing human BMP4 and GFP separated by an internal ribosome entry site, which is under the control of the human cytomegalovirus (CMV) promoter. BMP4 and GFP will be expressed individually with BMP4 secreted and GFP remaining inside the cells for tracking. This detailed construction was published previously [16, 18] and the transduction process was also previously described [22]. BMP4/GFP-transduced MDSCs (MDSCs/BMP4/GFP) were sorted using  fluorescence-activated cell sorting (FACS) for GFP after expansion. The BMP4 secretion level in the supernatant was measured using human BMP4 Quantikine ELISA Kit (DBP400, R&D system). The BMP4 secretion level was converted to ng/million cells/24 h for all populations as previously described [22].

### Osteogenic differentiation using pellet culture for MDSCs/BMP4/GFP

Osteogenic differentiation using three-dimensional (3D) pellet culture for young and old MDSCs/BMP4/GFP was performed following a previously published protocol [24]. Four pellets were set up using 2.5 × 10^5^ MDSCs/BMP4/GFP/pellet. Osteogenic medium was changed 3 times a week, osteogenic differentiation was terminated at 4 weeks after culture in osteogenic medium. The osteogenic differentiation was evaluated using microcomputed tomography (Micro-CT; Viva CT 40; Scanco Medical, Scanco USA Inc., Wayne, PA, USA). The pellets were scanned in PCR tubes with Micro-CT using the following settings: 70 kVp of energy, 112 µA, 200 ms integration time, an isotropic voxel size of 21 µm and medium resolution. The mineralized pellet volume was evaluated with Micro-CT 3D quantification software using the following parameters: Gauss Sigma 0.8, Gauss support 1.0, and threshold 122.

### Characterization of a dose-dependent effect of BMP4 on the proliferation, mitochondria biogenesis and osteogenic differentiation of young and old MDSCs after single and double transduction

Young and old MDSCs (un-transduced, single and double transduction) were cultured and passaged. Initial cell number at the time of seeding and the cell yield at time of next passage were analyzed to determine the cell population doubling time [36, 37]. Cell proliferation was also analyzed using Ki67 immunofluorescent staining using mouse anti-Ki67 antibody (9449S, Cell Signaling Technology, 1:500 dilution). To test mitochondrial function, six populations of cells were seeded in a 24-well plate with four replicates at 4 × 10^3^/well. Cells were allowed to grow for 3 days and then labeled with LDS-751 at 0.2µg/ml and Hoechst 33342 (1 µg/ml) for nuclear staining as previously reported because LDS-751 labeled mitochondria not nuclear [40]. The fluorescence intensity was measured at 543 nm excitation/712 nm emission  (LDS-751) and 350 nm excitation/462 nm emission (Hoechst 33342) using the Infiniti Pro 200 plate reader (Tecan). In addition, Mitotracker Red FM (Thermofisher Scientific) was used to test mitochondrial oxidation and was performed using 0.5 µM Mitotracker Red FM and 1 µg/ml Hoechst 33342. The fluorescence intensity was measured at excitation/emission wavelengths of 581 nm/644 nm  (Mitotracker Red FM) and 350 nm/461 nm (Hoechst 33342), respectively. Mitotrackers’ labeling for mitochondria and nuclear staining was performed in Dulbecco′s Phosphate Buffered Saline (DPBS) for 15 min at 37 °C. After labeling, cells were rinsed twice with DPBS and resuspended in 0.5 ml DPBS for measuring fluorescence intensity. Immediately after reading, for the Mitotracker Red FM plate, DPBS was removed, and 0.5 ml complete PM was added to each well. Fluorescent images were taken using Nikon Eclipse Ti2 inverted microscope using the red channel and blue channel for Hoechst 33342.

Furthermore, osteogenic differentiation using monolayer culture in osteogenic medium, was also performed in young and old MDSCs after single and double BMP4/GFP transduction. Un-transduced young and old MDSCs were not performed because they need BMPs to undergo osteogenic differentiation. After 5 days of osteogenic differentiation, cells were lysed in TRIzol reagent, RNA was extracted and Q-PCR was performed for *Runx2*, *Osx*, *Alp* and *osteocalcin* (*Ocn*) as stated above. Gene expression was first normalized by calculating Delta-Cycle of threshold (Delta-CT) by subtracting the CT value of GAPDH. The young single BMP4/GFP-transduced MDSCs were used as control (theoretic value should be 1), and the gene expression of each group was compared and reported using fold changes (2^-Delta-Delta-CT^).

### Critical sized skull defect and cell transplantation

Male CD-1 nude mice (Charles River) (*N* = 6 for each group, 6 weeks old) were used for this study. The creation of critical sized calvarial bone defect followed a previously described protocol [19]. Mice were anesthetized using 2% isoflurane, and an incision was made just off the midline of the skull on the scalp, and the right parietal bone was exposed. After removal of the periosteum, a 5-mm bone defect was created using a 5-mm diameter trephine drill (Fine Science Tools). The bone was carefully removed, and the defect area was rinsed with normal saline. 5 × 10^5^ culture expanded MDSCs/BMP4/GFP were re-suspended in 10 µl PBS and mixed with 20 µl thrombin immediately before their transplantation into the defect area of the skull. Then 20 µl of fibrin sealant protein (Tisseel, Baxter) was placed on top of the cells and allowed to solidify for 1–2 min. The wound was closed with sutures. After mice recovered, they were given food and water ad libitum*.* Bone regeneration was monitored using Micro-CT.

Additionally, one population of young MDSCs/BMP4/GFP and old MDSCs/BMP4/GFP with relatively lower expression of BMP4 levels were re-transduced with another round of retroviral-BMP4/GFP to increase BMP4 expression levels. These double transduced MDSCs were further transplanted into critical sized calvarial defect as above and compared to single transduced young and old MDSCs/BMP4/GFP for their bone regenerative potential in vivo (*N* = 6 male nude mice/group). Micro-CT live scanning was performed as described below and sacrificed at 6 weeks after surgery.

### Live Micro-CT scanning and analysis

To monitor the skull defect healing in the CD-1 nude mice, live Micro-CT scans (Viva CT—40, Scanco Medical, Switzerland) were performed at day 1, and 2, 4, and 6 weeks after cell transplantation using 70kVP, 112 µA and 30 μm voxel size settings. To quantify the bone volume using Scanco evaluation software, the newly formed bone area was contoured in every slice. Three-dimensional bone volume was automatically generated by a 3D evaluation program using Gauss σ 0.8, Gauss Support 1, and the threshold at 163.

### Tissue decalcification, processing, paraffin embedding and sectioning

Mice were sacrificed at 6 weeks after cell transplantation. Skull tissues containing the defect were harvested at 6 weeks after cell transplantation, fixed in NBF for 1 week, and then decalcified using 10% ethylenediaminetetraacetic acid disodium dihydrate (EDTA) containing 1% sodium hydroxide for 4 weeks. Tissues were paraffin-embedded, and sections were cut into 5 μm thickness. H&E staining was performed to investigate the general morphology according to an online protocol (https://www.ihcworld.com/_protocols/special_stains/h&e_ellis.htm). Herovici’s staining was performed to reveal the formation of major bone matrix collagen 1 according to a published protocol [23, 24, 41].

### Immunohistochemistry of GFP to trace donor cells

Immunohistochemistry of GFP was performed as previously described [22]. Sections were deparaffinized in xylene and rehydrated through gradient ethanol to deionized H2O. After deparaffinization, the sections were washed three times in PBS and blocked with 5% normal donkey serum in PBS at room temperature for 1 h. Rabbit anti-GFP antibody (ab290, Abcam) was diluted 1:1000 in 5% normal donkey serum and applied to slides and incubated in 4 °C overnight. On secondary day, sections were washed with PBS for 5 min for three times. Slides were then immersed in 0.5% H_2_O_2_ in PBS to inactivate endogenous peroxidase for 30 min. After washing in PBS three times, the sections were incubated with goat anti-rabbit-biotin (BA 1000, Vector Laboratories, 1:300 dilution in PBS) for 2 h at room temperature. After the sections were again washed in PBS three times, the sections were incubated in ABC reagent (Vector Elite ABC kits) for 2 h at room temperature. The positive cells were revealed with DAB kit (SK-4100, Vector Laboratories) for 6 min. After gentle rinsing with water, slides were counterstained with hematoxylin QS (H-3404, Vector Laboratories) for 20 s and rinsed in running tap water for 10 min to blue nuclei. The sections were dehydrated through gradient ethanol, cleared with xylene and mounted with xylene mount medium.

### Histology analysis of bone regeneration

To investigate the early events of bone regeneration, young MDSCs/BMP4/GFP with single transduction as well as old MDSCs/BMP4/GFP with single and double transduction were transplanted into the skull defect of male CD-1 nude mice (*N* = 6/group, 6 weeks old) which were then sacrificed 10 days after transplantation. The skull tissues were harvested and embedded in NEG freezing medium (Thermofisher Scientific) and snap frozen in liquid nitrogen. Cryosections were cut to a 8 µm thickness for further histological staining using Alcian blue staining for cartilage (http://www.ihcworld.com/_protocols/special_stains/alcian_blue.htm) and Von Kossa staining for bone regeneration http://www.ihcworld.com/_protocols/special_stains/von_kossa.htm).

### Western blot analysis of P18 and P16 expression

To investigate how BMP4 regulates P18 and P16 after cells transplantations, newly regenerated tissues in the skull defect area at 10 days after transplantation was dissected after cryosection for histology, rinsed with PBS, minced with scissors, and then 1 × RIPA buffer (#9806, Cell signaling) supplemented with phenylmethylsulfonyl fluoride (PMSF, #8553, Cell Signaling Technology), phosphatase inhibitor cocktails 2 (P5726-1ML, Sigma) and 3 (P0044, Sigma) was added to each sample at the volume of 100 mg/ml). Tissues were first homogenized with Ika Works, 3,720,001, T 18 Ultra-Turrax Volume Range Disperser (IKA) to completely disperse the tissues and then sonicated 10 s. The homogenized tissues were then centrifuged at 14000 g for 10 min at 4 °C. Supernatants were transferred to new tubes and protein concentrations were measured with Pierce™ BCA Protein Assay Kit (Cat#: 23,227). 30 µg proteins were mixed with 4 × Laemmli Sample Buffer (#1,610,747, BioRad) with β-mercaptoethanol. Samples were denatured and loaded on 4–20% Mini-PROTEAN® TGX™ Precast Protein Gels (#4,561,093, BioRad) and run at 120 V for 1 h. The proteins on the gel were then transferred using Turbo-Transfer system using Trans-Blot Turbo Mini 0.2 µm PVDF Transfer Packs (#1,704,156, BioRad). Western blotting was then performed by rinsing the membrane for 10 min in Tris-base buffered saline with 0.1% tween 20 (TBST) and then blocked using 1 × Blocker™ FL Fluorescent Blocking Buffer (10X) (Thermofisher Scientific) for 1 h and then incubated with primary antibodies. For the P18 membrane, rabbit anti-P18 CDKN2C (1:500, PA5-99462, Thermofisher Invitrogen), goat anti-GFP (1:1000, ab6673, Abcam) and mouse anti-β-actin (1:3000, A5441, Sigma) were added in 8 ml blocking buffer. For the P16 membrane, mouse anti-P16Ink4A (1:200, SC1661, Santa-Cruz), goat anti-GFP (1:1000, ab6673, Abcam) and mouse anti-β-actin (1:3000, A5441, Sigma) were added. Primary antibodies were incubated overnight at 4 °C with agitation. After washing three times with TBST, membranes were incubated with fluorescent conjugated secondary antibodies for 1 h in TBST with agitation. For P18, Donkey anti-Goat IgG (H + L) Highly Cross-Adsorbed Secondary Antibody, Alexa Fluor Plus 488 (1:5000 dilutions in TBST), donkey anti-Mouse IgG (H + L) Cross-Adsorbed Secondary Antibody, DyLight 800 (1:10,000 dilution in TBST); Donkey anti-Rabbit IgG (H + L) Highly Cross-Adsorbed Secondary Antibody, Alexa Fluor 680 (1:5000 dilution in TBST) were added. For the P16 detection, donkey anti-Goat IgG (H + L) Highly Cross-Adsorbed Secondary Antibody, Alexa Fluor Plus 488 (1:5000 dilutions in TBST), Donkey anti-Mouse IgG (H + L) Cross-Adsorbed Secondary Antibody, DyLight 800 (1:10,000 dilution in TBST) were added. Donkey anti-mouse IgG binds to both β-actin and P16 for P16 membrane but with different band sizes. Membranes were then washed and imaged with ChemoDoc MP system (BioRad)  with optimized exposure time for each channel. Two membranes for each gene were available for quantification and only one membrane image was used for data presentation. Band intensities were quantified using BioRad Image Lab software. The ratio of target gene/GFP was presented.

### Statistical analysis

Sample number was determined using power analysis with alpha = 0.05 and 1-*β* = 0.80 (power). Analysis of variance (ANOVA) and Student t test was used to compare multiple groups or two groups using Graphpad Prism 9. *P* < 0.05 was considered to statistical difference.

## Results

### Old MDSCs demonstrated reduced cell proliferation and multipotent differentiation when compared to young MDSCs

**Fig. 1:**
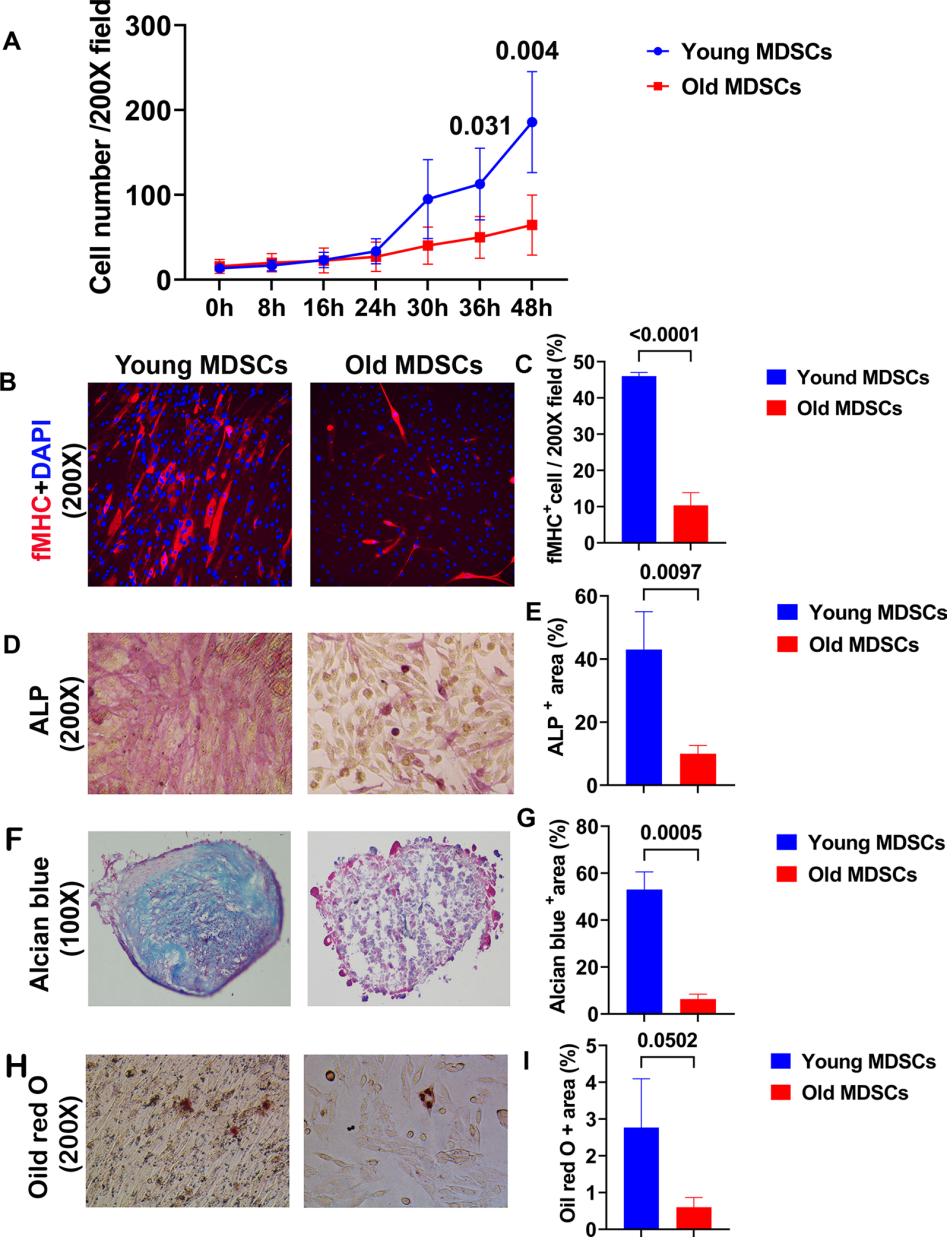
Old MDSCs demonstrated reduced cell proliferation and multipotent differentiation when compared to young MDSCs. **A** Proliferation of young and old MDSCs measured by live-cell imaging system. Old MDSCs showed decreased cell numbers at 36 and 48 h after seeding (*P* = 0.031 and 0.004, respectively). **B–C** Myogenic differentiation. fMHC positive cells were significantly decreased in old MDSCs compared to young MDSCs after 7 days differentiation (*P* < 0.0001). fMHC stained red and nuclei stained blue. **D–E** Osteogenic differentiation. ALP positive staining percentage was significantly lower in old MDSC compared to young MDSCs (*P* = 0.0097). **F–G** Chondrogenic differentiation using pellet culture. Alcian Blue positive area was significantly lower in pellets formed by old MDSCs compared to that of young MDSCs (*P* = 0.0005). **H–I** Adipogenesis. Oil red positive (dark red) area percentage was significantly lower in old MDSCs group compared to that of young MDSCs group (*P* = 0.0502)

Young and old MDSCs were analyzed for multipotent differentiation (myogenic, osteogenic, chondrogenic and adipogenic differentiation). The results indicate that old MDSCs exhibit reduced proliferation as revealed by cell numbers at different time points after cell seeding, when compared to young MDSCs (Fig. 1A). The myogenic differentiation marker, fast myosin heavy chain (fMHC) positive cell percentage, was significantly decreased in old MDSCs when compared to the young MDSC group (Fig. 1B–C). ALP staining and quantification showed the osteogenic differentiation was also significantly decreased in the old MDSC group when compared to the young MDSCs group (Fig. 1D–E). Alcian blue staining and quantification of blue matrix showed a decrease in chondrogenic differentiation of old MDSCs when compared to the young MDSCs (Fig. 1F–G). Furthermore, oil red staining and quantification demonstrated that the adipogenic potential of old MDSCs also was decreased compared to young MDSCs (Fig. 1H–I).

### Old MDSCs exhibit a greater percentage of cells in the G0/1 phase and higher P16 and lower P18 expression, when compared to young MDSCs

**Fig. 2:**
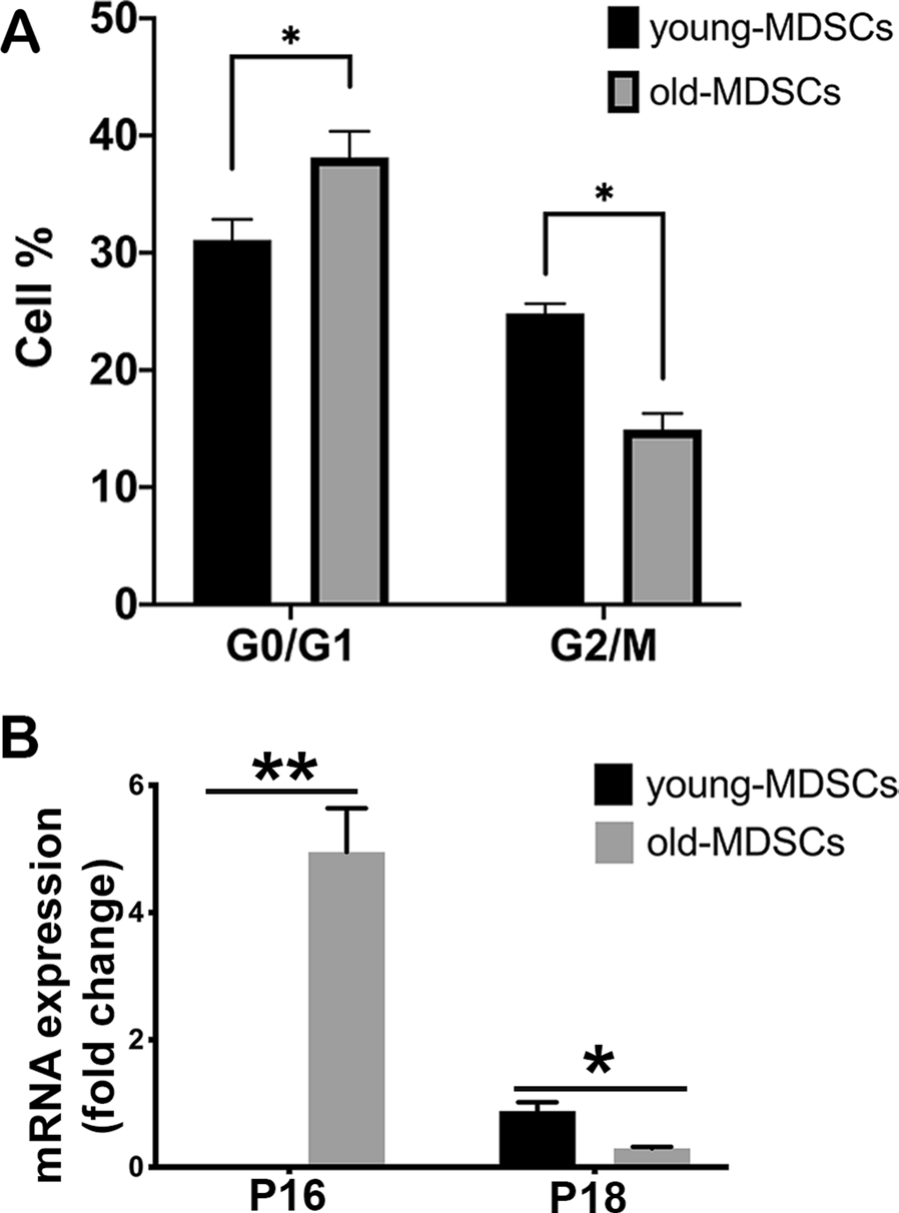
Old MDSCs exhibit a greater percentage of cells in the G0/1 phase and higher P16 and lower P18 expression, when compared to young MDSCs. **A** Propidium iodide (PI) staining of DNA detected by flow cytometry of young and old MDSCs. Percentage of cells in G0/1 phase is significantly higher while percentage of cells in G2/M is significantly lower in old MDSCs compared to young MDSCs (*P < 0.05). **B** qPCR analysis of P16 and P18. P16 mRNA expression is undetectable low in young MDSCs, but high in old MDSCs (**P < 0.01). P18 mRNA expression is significantly lower in old MDSCs than in young MDSCs (*P < 0.05)

Cell cycle analysis of young and old MDSCs was performed using propidium iodide staining and flow cytometry analysis and demonstrated that old MDSCs showed significantly higher G0/1 phase cell percentages and lower G2/M phase percentages cells (Fig. 2A). Q-PCR analysis revealed that old MDSCs expressed significantly higher level of P16 and a lower level P18 expression, when compared to young MDSCs (Fig. 2B).

### The osteogenic potential of old MDSCs remained significantly lower compared to young MDSCs, after BMP4 transduction

**Fig. 3:**
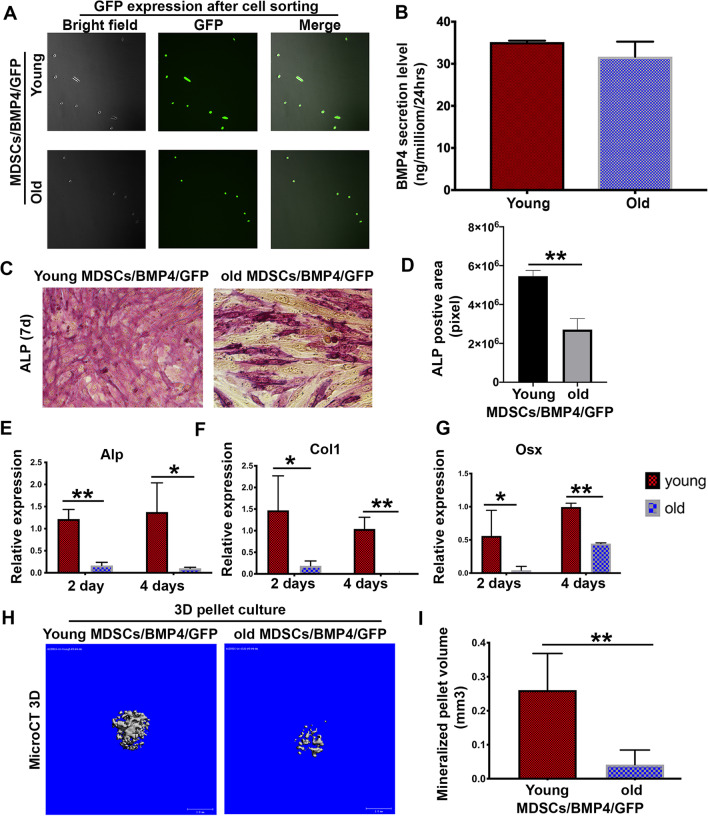
The osteogenic potential of old MDSCs remained significantly lower compared to young MDSCs, after BMP4 transduction. **A** Transduction efficiency of BMP4/GFP transduction for both young and old MDSCs. The efficiency of BMP4/GFP transduction on both young and old MDSCs is less than 100%. After cell sorting based on GFP positivity, GFP positive cells is at 100% for both young and old MDSCs. **B** BMP4 secretion level of young and old MDSCs after GFP cells sorting. No significant difference on the secretion levels of BMP4 between young and old MDSCs/BMP4/GFP. **C–D** Osteogenic differentiation of monolayer culture for young and old BMP4/GFP-transduced MDSCs. ALP positive staining area (purple pixel) is significantly lower in the old MDSC group compared to young MDSCs group. **P < 0.05. **E–G** qPCR analysis of Alp, Col1, and Osx.*P<0.05, **P<0.01. **H–I** MicroCT scan for osteogenic pellets of BMP4/GFP transduced MDSCs. Old MDSCs/BMP4/GFP could undergo osteogenic differentiation as demonstrated by mineralization using microCT scanning. However, the mineralized pellet volume of old MDSCs/BMP4/GFP was significantly smaller than young MDSCs/BMP4/GFP-transduced despite expressing similar levels of BMP4 at around 30 ng/million cells/24 h.**P < 0.01

To further investigate whether BMP4 transduction improves the osteogenic potential of old MDSCs, young and old MDSCs were transduced with a bone morphogenetic protein 4/green fluorescent protein (BMP4/GFP) retrovirus. 48 h after transduction, GFP was detected in BMP4/GFP-transduced MDSCs but less than 100% (data not shown). After GFP sorting, the sorted cells were cultured in the PM and both young and old BMP4/GFP-transduced MDSCs were found to be 100% GFP positive (Fig. 3A), indicating that all cells expressed BMP4. Both young and old BMP4/GFP-transduced MDSCs secreted similar amounts of BMP4 (around 30 ng/million cells/24 h) (Fig. 3B). Osteogenic differentiation based on alkaline phosphatase (ALP) staining (86CKit, Sigma) revealed that old MDSCs showed significantly lower ALP-positive area percentage (purple pixel) when compared to young MDSCs despite expressing similar levels of BMP4 after BMP4/GFP transduction (Fig. 3C–D). Next, expression of other osteogenic markers was quantitated using qPCR analysis for monolayer osteogenic culture. Alp, Collagen 1 (COL1) and osterix (Osx) expression levels were found to be significantly lower in the old MDSCs after day 2 and day 4 in osteogenic differentiation medium when compared to young MDSCs (Fig. 3E–G). 3D pellet culture for osteogenic differentiation of BMP4/GFP-transduced young and old MDSCs was performed and demonstrated that old BMP4/GFP-transduced MDSCs could undergo osteogenic differentiation as demonstrated by micro-CT, but the mineralized pellet volume was significantly lower than in the young BMP4/GFP-transduced MDSCs (Fig. 3H–I).

### The bone regenerative capacity of old MDSCs is inferior to young MDSCs despite expressing similar level of BMP4

**Fig. 4:**
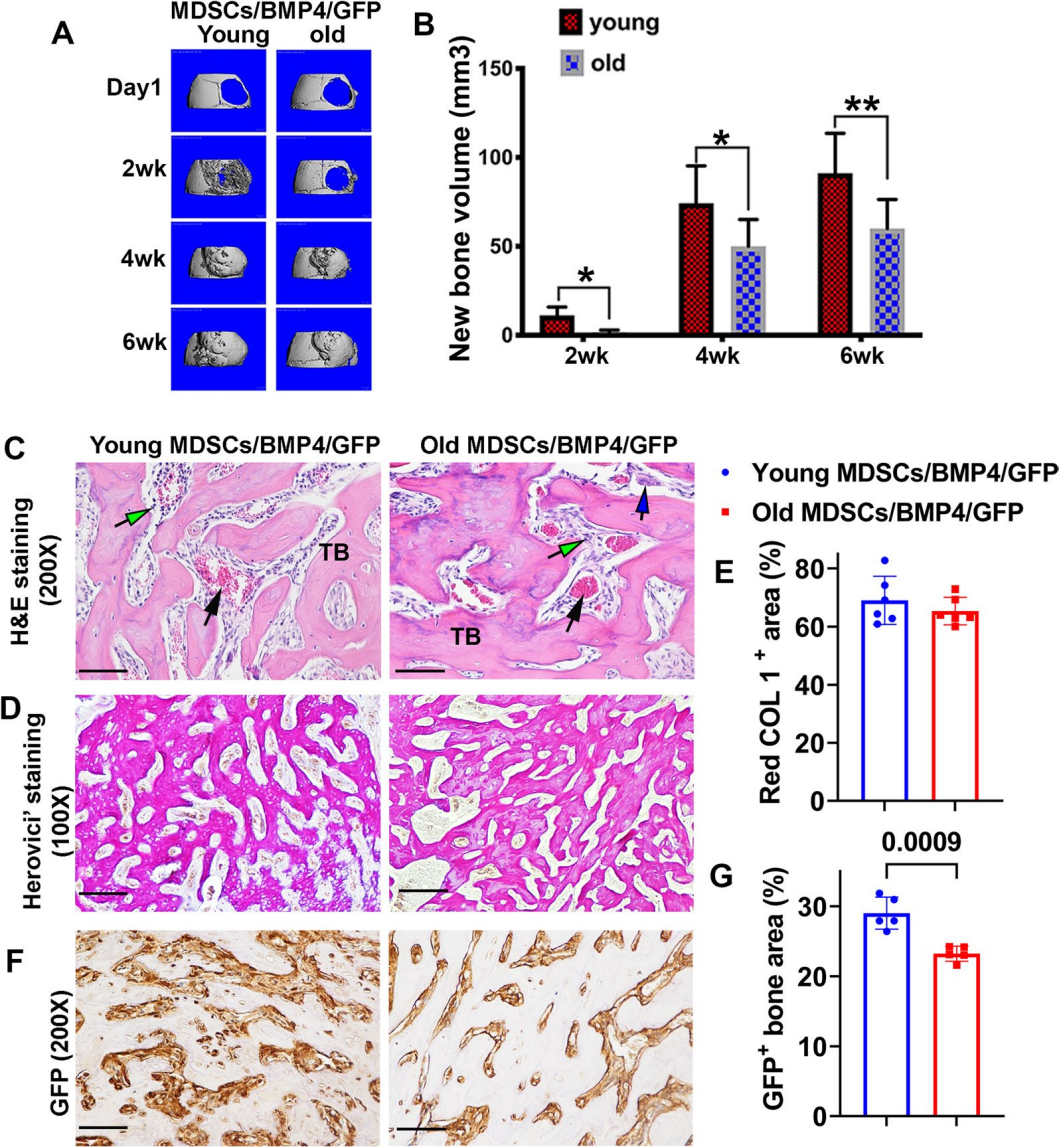
The bone regenerative capacity of old MDSCs is inferior to young MDSCs despite expressing similar levels of BMP4. **A** Micro-CT 3D images at different time points after cell transplantation. **B** Quantification of new bone volume in the skull defect at different time points showed significantly less new bone formed by old MDSCs/BMP4/GFP compared to young MDSCs/BMP4/GFP in the skull defect area when both young and old MDSCs/ BMP4/GFP have BMP4 secretion level of around 30 ng/million cells/24 h. *P < 0.05, **P < 0.01. **C** H&E staining for the new bone on the skull defect. The new bone is mainly trabecular bone (TB) constituted of new bone matrix and bone marrow including myeloid cells (green arrows, red blood cells (black arrows) and megakaryocytes (Blue arrows). No significant differences between young and old MDSCs/BMP4/GFP groups were found. Scale bars = 200 µm. **D–E** Herovici’s staining for the new bone on the skull defect and quantification. COL1 stained red/pink color. COL3 3 stained blue color. The newly formed bone tissues showed cancellous bone structure in both groups. Quantification of COL1 area percentage showed no significant difference between young and old MDSCs/BMP4/GFP groups. Scale bars = 200 µm. **F–G** Immunohistochemistry of GFP to trace donor cells for the new bone on the skull defect with associated quantification. The new bone area showed that most of the osteoblasts and osteocytes are GFP positive in both groups. Quantification for GFP positive area of the new bone on the skull defect showed lower GFP^+^ area percentage in the newly regenerated bone in old MDSCs/BMP4/GFP group compared to young MDSCs/BMP4/GFP group. *P* = 0.0009. Scale bars = 100 µm

Micro-CT results showed young BMP4-transduced MDSCs nearly completely healed the 5 mm calvarial bone defect at 2 weeks after transplantation while the old MDSCs showed incomplete healing of the bone defect at this time point. However, by 4 and 6 weeks post implantation, both the old and young BMP4/GFP-transduced MDSCs showed complete healing of the bone defect (Fig. 4A). Quantification of the bone volume indicated significantly less bone formed by the old BMP4/GFP-transduced MDSCs when compared to young MDSCs at the 2-week time point (Fig. 4B). The newly regenerated bone volume of the old MDSCs was still significantly lower at 4 and 6 weeks after cell transplantation when compared to young MDSCs, although the defects were also completely healed (Fig. 4B).

### Old MDSCs formed functional bone when transduced with BMP4/GFP despite a decrease in new bone volume

H&E staining revealed the new regenerated bone in the old MDSC group showed similar cancellous bone architecture, as indicated by the formation of bone matrix and bone marrow, when compared to the young MDSC group (Fig. 4C). Herovici’s staining was performed and demonstrated red/pink bone matrix COL1 constituting the majority of the newly formed bone in both groups. No significant differences in morphology and COL1 area percentage were found between the young and old transplanted-MDSC groups (Fig. 4D–E). Immunohistochemistry staining showed that GFP-positive donor cells constituted the majority of the osteoblasts and osteocytes in the new regenerated bone in both the young and old MDSCs/BMP4/GFP groups. This confirmed that the newly generated bone was mainly derived from transplanted MDSCs/ BMP4/GFP regardless of donor cell age (Fig. 4F). However, GFP^+^ area percentage in Old MDSCs/BMP4/GFP group is significantly lower than that of young MDSCs/BMP4/GFP group (*P* = 0.0009) (Fig. 4G).

### Increased BMP4 expression level in old MDSCs improve their bone regenerative potential to a level comparable to young MDSCs

**Fig. 5:**
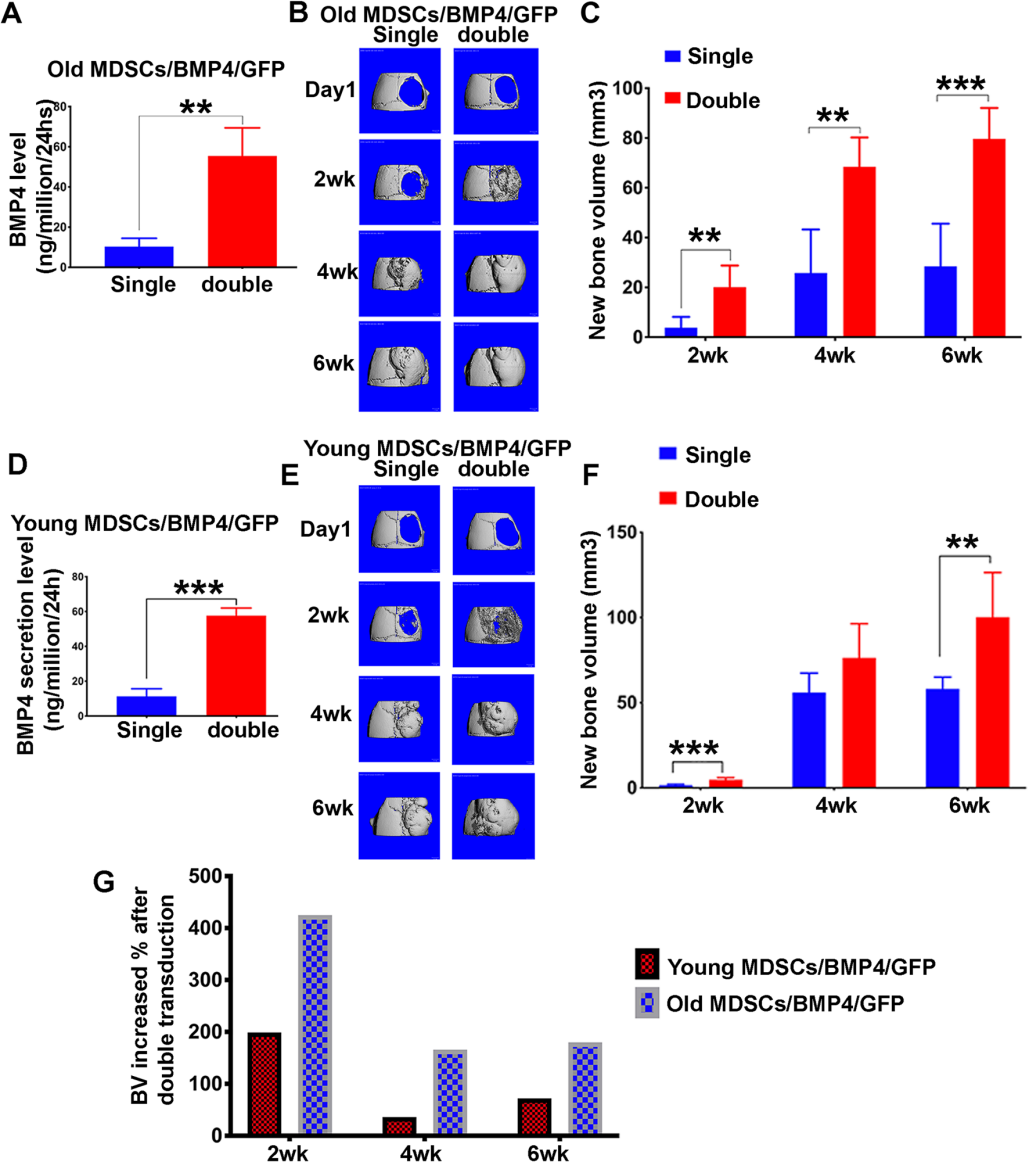
Increased BMP4 expression levels in old MDSCs improve their bone regenerative potential to levels comparable to young MDSCs. **A** BMP4 secretion levels after double transduction. **P < 0.01. **B** Micro-CT 3D images at different time points of single and double transduction of old MDSCs/BMP4/GFP. Bone formation enhancement is more obvious at the 2 weeks timepoint. Both groups showed complete defect healing at 4 weeks with similar bone density for both groups. **C** Quantification of new bone volume. **P < 0.01. ***P < 0.001. **D** BMP4 secretion level of single and double transduction of young MDSCs/BMP4/GFP. ***P < 0.001. **E** Micro-CT 3D images at different time points. Double transduction showed obvious accelerated bone regeneration at two weeks and nearly completely healed defects at 2 weeks after cell transplantation and complete healing of defect at 4 and 6 weeks after cell transplantation. **F** Quantification of new bone volume at different time points. At the 2 and 6 weeks timepoints, double transduction significantly increased new bone formation, but not at 4 weeks. **P < 0.01, ***P < 0.001. **G** Bone regeneration increase percentage after double transduction of young and old MDSCs/BMP4/GFP at different time points. Old MDSCs/BMP4/GFP showed an increase of 400% versus 200% at 2 weeks in young MDSCs/BMP4/GFP group and more than 150% at 4 and 6 weeks compared to less than 100% in young MDSCs/BMP4/GFP group after double transduction

To test whether increased expression level of BMP4 can further improve the bone regeneration capacity of old MDSCs, the FACS sorted retro-BMP4/GFP-transduced MDSCs (single transduced) that secrete low levels of BMP4 (around 10 ng/million cells/24 h) were re-transduced to increase BMP4 expression. After re-transduction (double transduced), the secretion levels of BMP4 increased to around 60 ng/million cells/24 h. 5 × 10^5^ culture-expanded BMP4/GFP single and double transduced old MDSCs were transplanted into the skull defect area of male CD-1 nude mice at an age of 6 weeks (Charles River) (Fig. 5A). As a result, as the secretion level of BMP4 increased in double transduced old MDSCs, there was a dramatic increase in bone regenerated at 2 weeks, and, consequently, the bone defect almost completely healed at 2 weeks, while more new bone formation was also detected at 4 weeks and 6 weeks. The quantification of the bone volume indicated that increased BMP4 expression levels in the old MDSCs significantly increased the new bone volume at all time points (Fig. 5B–C). It also demonstrated that the bone regeneration impairment of old MDSCs could be rescued by increasing BMP4 levels. Young MDSCs expressing a higher level of BMP4 also showed an increase in regenerated bone volume compared to young MDSCs expressing lower BMP4 levels (Fig. 5D–F); however, the difference was not significant at 4 weeks (Fig. 5F). These results demonstrated that BMP4 plays a critical role in MDSC-mediated bone regeneration. By calculating the percentage of new bone volume increases after BMP4/GFP double transduction for young and old MDSCs, with BMP4 secretion increasing from 10 to 60 ng/million cells/24 h, the percentage increase of bone volume in old MDSCs (400%) was found to be significantly higher than in young MDSCs (200%) at 2 weeks. The increase in bone volume was also higher in old MDSCs when compared to young MDSCs at 4 and 6 weeks (Fig. 5G). In fact, old MDSCs following double transduction regenerated more bone at 2 weeks (20.4 ± 8.71 vs. 4.73 ± 1.33, *P* = 0.002) after transplantation and similar amount of newly formed bone at 6 weeks compared to their young MDSC counterpart (79.6 ± 12.47 vs. 100.23 ± 26.2 mm^3^, *P* = 0.14). These results indicate that age-related bone regeneration impairment or decline in old MDSCs can be rescued by enhancing BMP4 expression.

### BMP4 transduction reverses abnormal CDK inhibitors expression of old MDSCs and function

**Fig. 6:**
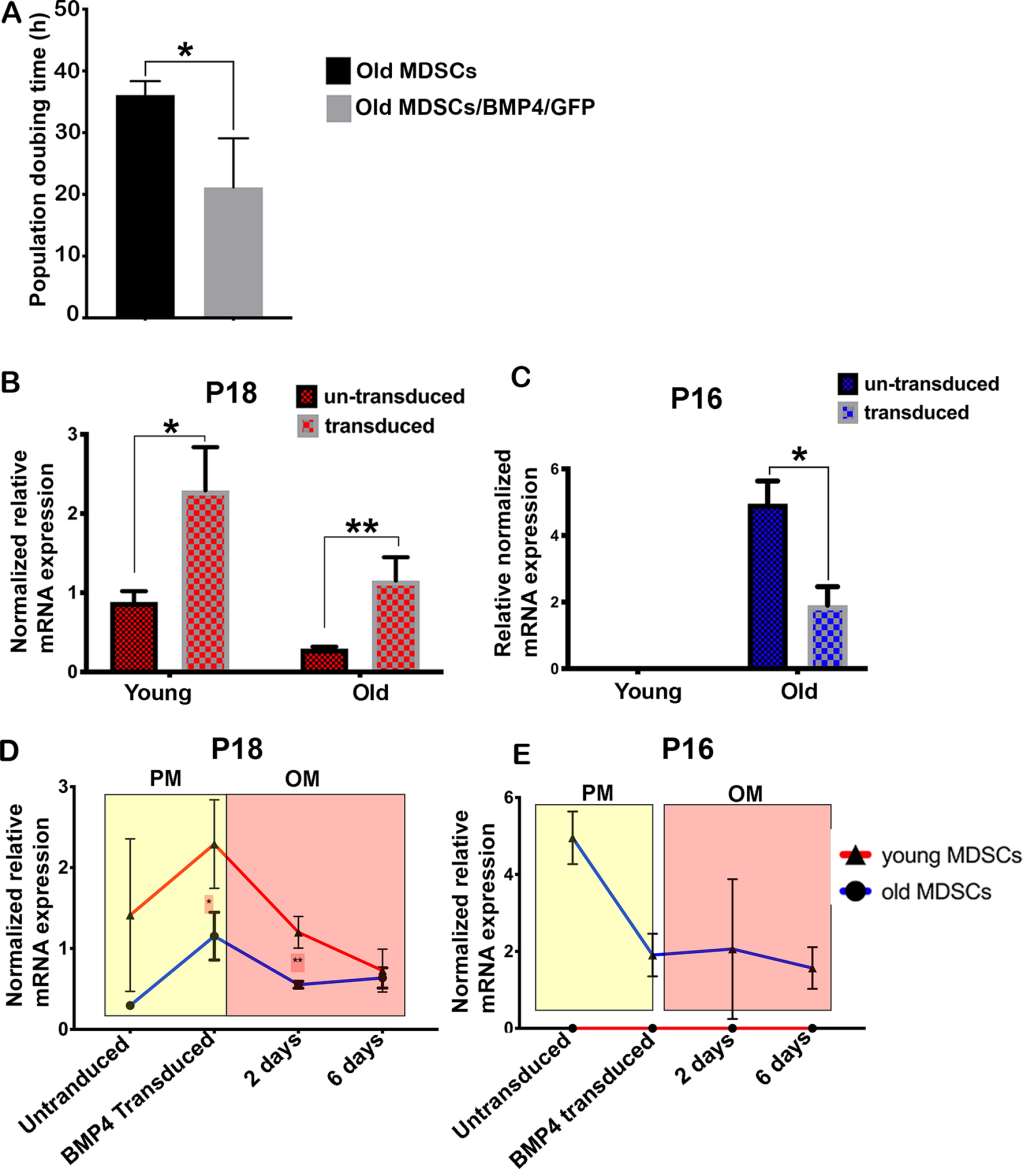
BMP4 reverse aged-related decline in cell proliferation via regulating P18 and P16 after single BMP4/GFP transduction. **A** Doubling time for old MDSCs before and after BMP4 transduction. BMP4/GFP transduction decreased the population doubling time of old MDSCs. *P < 0.05. **B** qPCR for P18 in transduced and un-transduced MDSCs. BMP4 transduction significantly increased P18 expression for both young and old MDSCs. *P < 0.05, **P < 0.01. **C** Q-PCR for P16 in BMP4 transduced and un-transduced MDSCs. Young MDSCs did not express detectable P16 before and after BMP4 transduction. BMP4 transduction significantly decreased P16 expression in old MDSCs. *P < 0.05. **D** QPCR for P18 in transduced and un-transduced MDSCs cultured in proliferation medium or osteogenesis medium. *P < 0.05, **P < 0.01. **E** Q-PCR for P16 in transduced and un-transduced MDSCs cultured in proliferation medium or osteogenesis medium

Since increasing BMP4 levels were found to rescue the impairment of in vivo bone formation of old MDSCs, it was further investigated as to whether BMP4 transduction also affected cell proliferation and differentiation. Doubling time was calculated based on the initial cell number, the final cell number, and the duration of culture. It was determined that the doubling time of old MDSCs was significantly decreased after BMP4/GFP transduction, which indicated that cell proliferation was increased (Fig. 6A). Furthermore, P18 mRNA expression was found to be significantly increased in both young and old MDSCs after BMP4 transduction (Fig. 6B). P16 mRNA expression was lower and undetectable in young MDSCs before and after BMP4 transduction. However, its expression was significantly decreased after BMP4 transduction in old MDSCs (Fig. 6C). To track the dynamic changes of P18 and P16 during cells expansion and osteogenic differentiation, the P18 and P16 expression levels were compared in different conditions. P18 mRNA expression increased after BMP4 transduction without osteogenic differentiation (yellow) but decreased after osteogenic differentiation (orange) in both young and old MDSCs. P18 expression is significantly lower in old MDSCs when compared to young MDSCs after BMP4/GFP transduction (*P* < 0.05), and during early osteogenic differentiation at 2 days (*P* < 0.01). However, there is no difference in P18 expression between young and old BMP4/GFP-transduced MDSCs at 6 days after differentiation in osteogenic medium (Fig. 6D). P16 mRNA expression decreased after BMP4 transduction and maintained a lower expression level in old MDSCs, whereas young MDSCs maintained undetectable levels of P16 expression before transduction, after BMP4/GFP transduction, and during osteogenic differentiation (Fig. 6F).

### Effect of increasing BMP4 level by double transduction on cell proliferation, mitochondrial function, and osteogenic differentiation in vitro

**Fig. 7:**
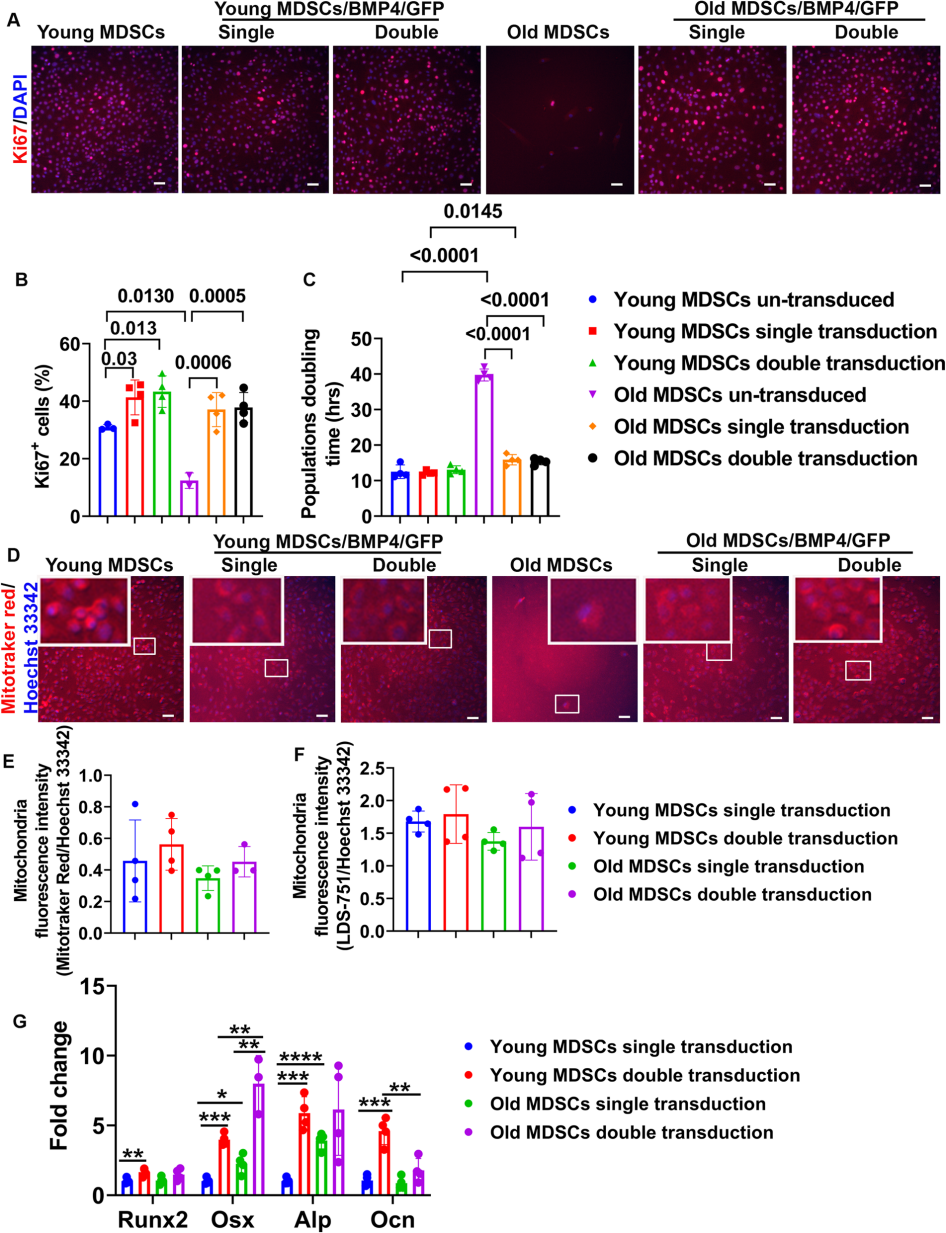
Dose-dependent effect of BMP4 after double transduction on cell proliferation, mitochondria function and osteogenic differentiation. **A**, **B** Ki67 staining and quantification. Ki67^+^ stained red in nuclei. Scale bars = 200 µm. Exact values are shown between group comparisons. **C** Population doubling time quantification. Exact values are showed between group comparisons. **D** Live mitochondrial staining with Mitotracker Red FM to show oxidation activity of mitochondria. Mitochondria are stained red (Mitotracker Red) and nuclei are stained blue with Hoechst 33342. Insets are the enlarged boxed area of cells to better display mitochondria in the cytoplasm. Scale bars = 200 µm. **E** Quantification of Mitotracker red fluorescence intensity relative to cell number using Hoechst 33342. **F** Quantification of LDS-751 fluorescence intensity relative to cell number using Hoechst 33342. **G** Q-PCR analysis of osteogenic differentiation of single and double transduced young and old MDSCs. *P < 0.05, **P < 0.01, ***P < 0.001. ****P < 0.0001

Two approaches were used to investigate the effect of double BMP4/GFP transduction on cell proliferation: analysis of Ki67 staining and population doubling time. Ki67 staining demonstrated significantly lower percentage of Ki67^+^ cells in old MDSCs compared to young MDSCs (*P* = 0.013). A higher percentage Ki67^+^ cells were found after single and double transduction of BMP4/GFP in young cells (*P* = 0.03 and 0.013, respectively) compared to un-transduced young MDSCs. Single and double transduction of old MDSCs showed significantly higher percentage of Ki67^+^ cell than un-transduced old MDSCs (*P* = 0.0006 and 0.0005, respectively). No statistical differences of the percentage of Ki67^+^ cells between single and double transduction for either young or old MDSCs were identified (Fig. 7A–B). As for population doubling time, un-transduced old MDSCs showed significantly longer population doubling time (*P* < 0.0001) than un-transduced young MDSCs. Single and double transduction did not significantly change the population doubling time of young MDSCs. However, single and double transduction significantly shortened the population doubling time of old MDSCs (*P* < 0.0001 for both). No statistical differences were found between double and single transduction for old MDSCs in terms of population doubling time. In addition, the population doubling time of old MDSCs with single transduction was still significantly longer than young MDSCs with single transduction (*P* = 0.0145). However, there was no statistical difference between young and old MDSCs after double transduction in population doubling time (Fig. 7C).

To test mitochondrial function, Mitotracker Red FM was used to label mitochondria. Mitochondria were labeled red after incubating with Mitotracker Red FM for 15 min. Fluorescent images showed Mitochondria stained red (Mitotracker Red) in the cytoplasm of the cells and nuclei stained blue with Hoechst 33342. There were no significant differences observed between double transduced, single transduced and un-transduced young MDSCs. Very few cells were observed in un-transduced old MDSCs due to very low proliferation rate. No obvious mitochondrial labeling difference was found between double transduced and single old MDSCs (Fig. 7D). Fluorescence intensity of mitochondria showed double transduction slightly increased relative fluorescence intensity (Mitotracker Red/ Hoechst 33342 ratio) for both young and old MDSCs, but no significant differences were observed between the groups. Un-transduced young MDSCs were not included because the cells had overgrown and peeled off from the culture plates to make the analyses not reliable. Old MDSCs showed very low level of fluorescence intensity due to a paucity of cells and was also not included in the quantification (Fig. 7E). Similarly, quantification using another mitochondrial fluorescent dye LDS-751 also showed mitochondrial fluorescence intensity (LDS-751/Hoechst 33342) was not significantly different among the different cell groups tested (Fig. 7F).

To investigate if double transduction will further increase osteogenic differentiation in vitro, osteogenic gene expression was analyzed using Q-PCR. The results indicated that double transduction significantly increased Runx2 expression (*P* < 0.01) in young MDSCs compared to young MDSCs after single transduction. Double transduction also increased Runx2 expression in old MDSCs but did not reach statistical significance (Fig. 7G). Os*x* expression was significantly increased by double transduction compared to single transduction for both young and old MDSCs (*P* < 0.001 and *P* < 0.01, respectively). Remarkably, old MDSCs after both single and double transduction expressed Osx in a significantly higher level than their young MDSCs counterparts (*P* < 0.05 and *P* < 0.01, respectively) (Fig. 7G). Double transduction significantly increased Alp expression when compared to single transduction of young MDSCs (*P* < 0.001). Double transduction also increased Alp gene expression of old MDSCs but this increase did not reach to statistical difference level. However, old MDSCs with single transduction expressed significantly higher Alp levels when compared to single transduced young MDSCs (*P* < 0.0001). The Alp expression in the double transduced old MDSCs was similar to the double transduced young MDSCs (Fig. 7G). Ocn gene expression (mature osteoblast marker) was significantly increased after double transduction when compared to the single transduction in young MDSCs (*P* < 0.001). Ocn was also increased after double transduction in old MDSCs but was not statistically significant. However, Ocn expression in old MDSCs was still significantly lower than the young MDSCs after double transduction (*P* < 0.01) (Fig. 7G).

### Regulation of P18 and P16 by BMP4 in vivo at early stage of bone regeneration

**Fig. 8:**
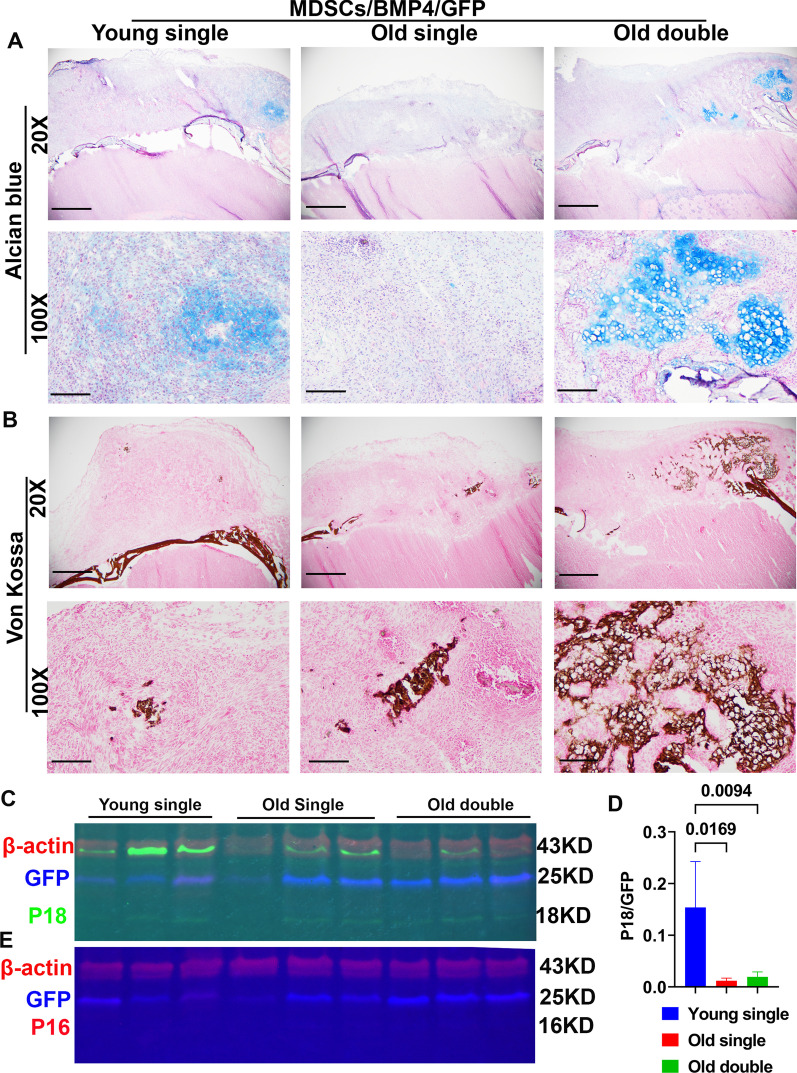
Bone regeneration at day 10 after in vivo transplantation of young and old BMP4/GFP-transduced MDSCs. **A** Alcian blue staining. Chondrocytes and cartilage stained blue. 20X magnification shows the entire defect area. Transplanted cells proliferated and formed thick cell layers on top of the critical sized defect. Old MDSCs with double transduction showed early endochondral bone formation at day 10 after transplantation with obvious chondrogenic nodule in blue as did young MDSCs. Scale bars = 1 mm for 20× and 200 µm for 100×, respectively. **B** Von Kossa Staining. Bone stained brown-black. Both young and old BMP4/GFP transduced MDSCs showed mineralization, old MDSCs with double BMP4/GFP transduction showed more mineralization than single transduction. These findings indicate that higher levels of BMP4 reversed the impairment of bone regeneration of old MDSCs. Scale bars = 1 mm for 20× and 200 µm for 100×, respectively. **C** Fluorescent western blot for P18 (green) and GFP (blue) with β-actin (red) as loading control. There is a green non-specific band in the P18 channel merging with the β-actin (red) channel. **D** Quantification of P18 expression relative to GFP showed old MDSCs/BMP4/GFP expressed less P18 compared to young MDSCs/BMP4/GFP, while increased BMP4 expression by double transduction slightly increased P18 expression. **E** Western blot of P16 (Red) and GFP (blue) and loading control β-actin (red) at day 10 after cell transplantation. P16 is not detectable by western blot at this time point of bone regeneration in both young and old MDSCs/BMP4/GFP group with single or double transduction

Alcian blue staining was performed at 10 days after cell transplantation to investigate early events of bone regeneration. Old MDSCs transduced with BMP4 formed chondrogenic nodules more slowly than young MDSCs expressing similar BMP4 levels. However, old MDSCs expressing higher levels of BMP4 (double transduction) formed hypertrophic chondrocytes at the same timepoint (Fig. 8A). Von Kossa staining showed that both young MDSCs and old MDSCs with single transduction formed focal mineralization at day 10, while old MDSCs with double transduction formed significantly more mineralized bone at this timepoint indicating higher expression levels of BMP4 can reverse bone regeneration defects in older MDSCs (Fig. 8B). Western blots analysis showed P18 expression in all three groups (green bands) at 18KD region (Fig. 8C). Quantification of relative expression (P18/GFP) showed that young MDSCs express higher levels of P18 compared to old MDSCs with single or double BMP4/GFP transduction, but that double transduction slightly increased P18 expression (Fig. 8D). Furthermore, P16 expression was not detected in all groups (red bands at 16KD region). Only GFP (blue band) and β-actin band (red band at 42KD region) was detected on the P16 membrane (Fig. 8E).

## Discussion

The most important findings of this study are that chronologically old MDSCs exhibited slower cell proliferation and reduced multipotent differentiation capacity. The reduced cell proliferation of old MDSCs is related to increased P16 and decreased P18 expression. However, BMP4 transduction increased cellular proliferation in old MDSCs as revealed by the decrease in population doubling time. Old MDSCs transduced with BMP4/GFP could regenerate equivalent functional bone in critical sized bone defects (although relatively slower initially and contributed less to the new regenerated bone) during bone regeneration. Importantly, increased BMP4 expression levels for old MDSCs, after double transduction, further enhanced osteogenic differentiation in vitro*,* increased bone formation in vivo and was found to have similar amounts of regenerated bone compared to young MDSCs. This observation correlated with significant decreases in P16 and increases in P18 expression in old MDSCs after BMP4 transduction. Furthermore, increased BMP4 expression levels in old MDSCs accelerated bone regeneration of old MDSCs which also correlated with an increase in P18 expression and a decreased in P16 expression after cell transplantation in the calvarial bone defect.

Stem cell proliferation, multipotent differentiation, and tissue regeneration capacity are tightly regulated by cell cycle progression. It has been demonstrated that MDSCs from progeria mice lose regenerative potential during aging and can be rescued by the paracrine factors secreted by young MDSCs [13, 14]. However, which specific factors play roles are not clear and whether the cell cycle regulation can reverse old stem cell dysfunction is not known. This study sought to provide further understanding as to the mechanism of cell cycle regulation in MDSC aging and bone regeneration. This study demonstrates that old MDSCs exhibit significant reduction in their proliferation and multipotent differentiation capacity in vitro. More importantly this age-related decline in old MDSCs was found to correlate with increases in P16 and decreases with P18 when compared to young MDSCs. Previously, it has been reported cell cycle kinetics in the G1 phase are critically associated with the fate of adult stem cells [42]. P16 was found to increase with age, accumulate, and modulate specific age-associated stem cell functions [43], and as such increased expression of P16 is a hallmark of aging and cells senescence [44]. P18 was previously noted to balance the relative asymmetry between self-renewal and differentiation in young stem cells [33, 34]. Knocking out P18 has been shown to release hematopoietic stem cells from the G1 phase to enhance their ex vivo expansion and resulted in long-term engraftment in hematopoietic stem cell transplantation in irradiated mice [45, 46].

Interestingly, the current study revealed the proliferation of old MDSCs was significantly enhanced by BMP4/GFP transduction indicating that BMP4 may regulate cell cycle progression. Indeed, it was also found that P16 is significantly decreased and P18 is significantly increased in old MDSCs after BMP4 transduction. Furthermore, increased BMP4 expression after double transduction significantly increased *Osx* expression in old MDSCs to a level of Osx expression higher than their young MDSC counterpart. In addition, old MDSCs expressed similar levels of *Alp* compared to young MDSCs following double transduction. This finding implies that BMP4 may be a novel regulator of aging, especially in bone regeneration. It is known that BMP signaling not only induces bone and cartilage formation [47–49], but also plays multiple roles in stem cell functional regulation such as self-renewal [28, 29, 50–58]. In the model of Drosophila ovarian germline stem cells (GSCs), it has been demonstrated that compromised BMP signaling contributes to GSC aging and decreased lifespan [3]. It is therefore hypothesized that BMP signaling may promote MDSC proliferation via regulation of the cell cycle through downregulation of P16 and upregulation of P18, in addition to its basic function in promoting bone regeneration.

Furthermore, old MDSCs secreting similar BMP4 levels were found to still regenerate functional bone in vivo when transplanted into a critical size bone defect, although the resulting bone volume was relatively lower, and the bone regeneration process relatively slower compared to young MDSCs. Remarkably, increased BMP4 levels by double transduction of old MDSCs resulted in the regeneration of bone to a level equivalent to their young MDSCs counterpart. This finding further validates the role of BMP4 in attenuating/reversing the aging related decrease in the bone regenerative potential of old MDSCs.

The bone regeneration capacity increased more than 400% when the BMP4 level was increased from 10 to 60 ng/million cells/24 h in old MDSCs, while the bone volume young MDSCs increased to only 200% at 2 weeks after cells transplantation. These results indicate the important function of BMP4 on old MDSC function and bone regeneration. Previous studies have shown that MDSCs express BMP receptors (BMPRs), but there was no BMP4 secretion detected without BMP4/GFP transduction by ELISA, which indicates that MDSCs can react to BMP signaling from the microenvironment via autocrine or paracrine effects [22]. It has been shown that BMP4 treatment of embryonic stem cells maintain self-renewal via activated Id gene, which encodes negative bHLH factors, while overexpression of BMP4/Smad1/5 signaling inhibitors, Smad 6 and 7, resulted in reduced ES cell clones [29]. During the aging process, the gradual decline of P18 may interfere with the increasing demand for stem cell self-renewal. If the cell cycle blockade is eventually dominated by P16 in the G1 phase, inevitably, the cell will undergo senescence. The current study has demonstrated that BMP4 upregulates P18 in old MDSCs and delays the process of the cell cycle blockade being dominated by P16 and as a result is able to rescue the reduction in bone regeneration capacity of old MDSCs. However, how BMP4 regulates P16 and P18 expression is still unknown. On the other hand, it has been reported that BMP4 (10–50 ng/ml, a range also suitable for osteogenic differentiation) acts as an oxidative stress mediator and induced cellular senescence in retinal pigment cells via activation of pSmad1/5/8 and the p38 pathways [59]. Since we found that BMP4 transduction significantly increased proliferation and their bone regenerative potential of old MDSCs, we speculate that BMP4 may have a different effect in various cell types and microenvironment the cells exposed. Further studies are needed to address the different effect of BMP4 in various cell types.

It has been shown BMP7 could alleviate serial culture-induced cell senescence by increasing cell proliferation, telomerase activity, and G0/G1 phase of cells by down-regulating P16 and P53. The mechanism is via activation of PI3K/AKT pathway [60]. It has also been shown the BMP-pSMAD-ID signaling axis suppressed p16/INK4A-mediated cell senescence during fibroblast reprograming to produce induced pluripotent stem cells [61]. Further, it has been reported that adding BMP6 to prostate cancer cells inhibits cancer cell growth by activation P21, P18 and P19 expression in the cells [62]. Hence, the regulation of cell cycle especially P16 and P18 appears not only mediated by BMP4 but also by other BMPs. This indicates that BMPs may be potent factors for rejuvenating old stem cells via regulating cell cycle inhibitors.

Lastly, the current study revealed that increased BMP4 secretion levels accelerated bone regeneration as revealed by Alcian blue staining and Von Kossa staining. The P18 expression was maintained during bone regeneration despite its expression level being lower in the old MDSCs/BMP4/GFP group compared to the young MDSCs/BMP4/GFP group. This result confirms the important role of P18 in bone regeneration. Importantly, P16 expression was essentially blocked by BMP4 transduction during bone regeneration. Indeed, it has been shown that clearing P16 expressing stress-induced senescent cells with dasatinib and quercetin (D + Q) enhanced bone formation in a critical sized bone defect [63]. In a recent study, it was found that aged skeletal stem cell bone repair impairments could only be rescued by delivery of BMP2 and antagonist of monocyte colony stimulation factor [64].

## Conclusions

**Fig. 9:**
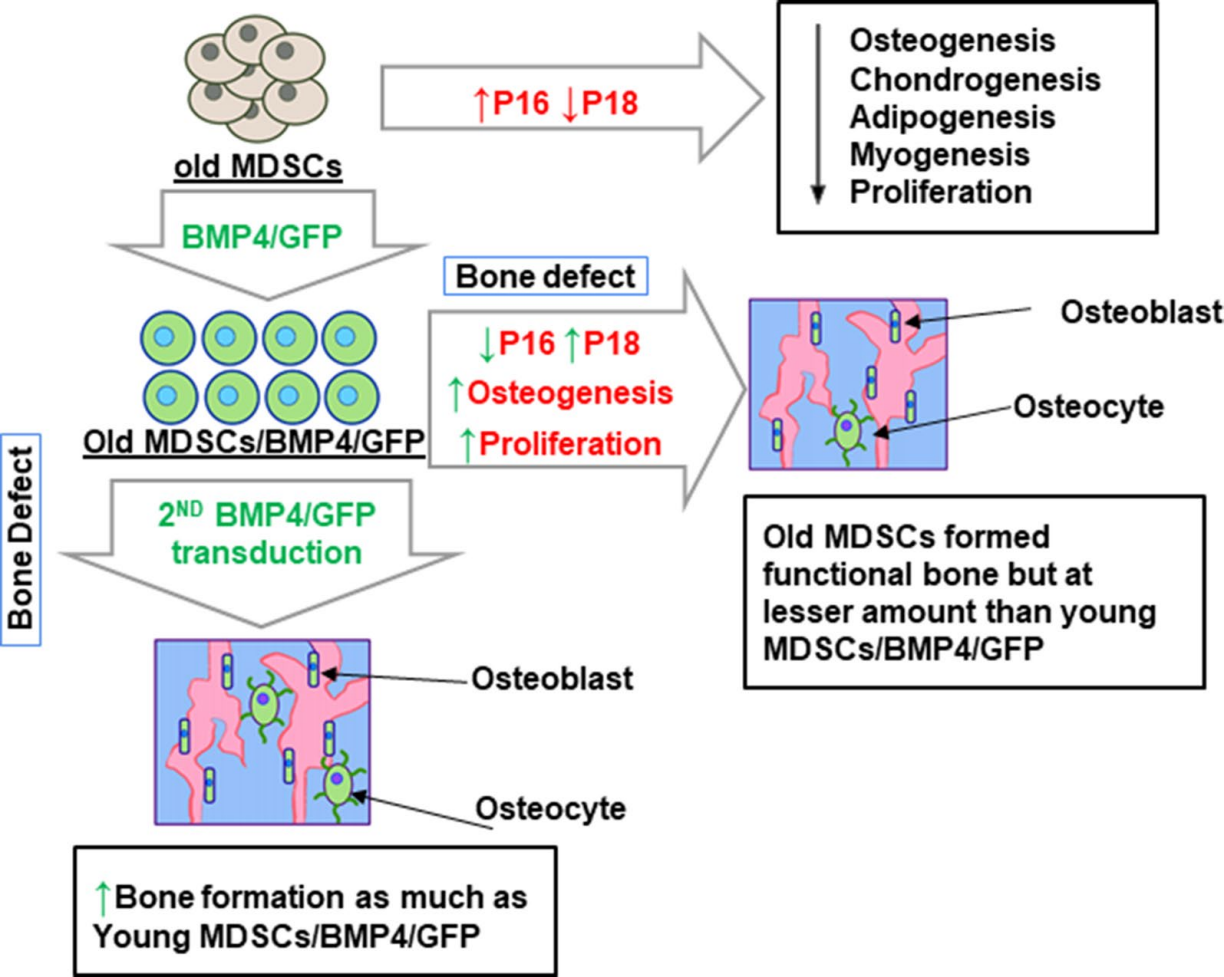
Schematic representation of the role of BMP4 on the regulation of stem cell function and bone regeneration

In summary, the current study’s results indicate that BMP4 transduction reverses the impairment of old MDSC in cell proliferation by up-regulating P18 and down-regulating P16 expression. Old MDSCs transduced with BMP4 could regenerate functional bone in vivo and increasing BMP4 expression levels can endow MDSCs bone regeneration capacity to a level comparable to young MDSCs through maintenance of P18 levels and through blocking P16 expression (Fig. 9). These findings represent a novel mechanism by which BMP4 can offset the aging defects on osteogenic differentiation in vitro and bone regeneration in vivo in old MDSCs.

## Data Availability

The datasets used and/or analyzed during the current study are available from the corresponding author on reasonable request.

## Abbreviations

MDSCs: Muscle-derived stem cells
P16: P16 Ink4A
P18: P18INK4C
BMP4: Bone morphogenetic protein 4
GFP: Green fluorescent protein
CDK: Cyclin-dependent kinase
GSCs: Germline stem cells
EDTA: Ethylenediaminetetraacetic acid disodium dihydrate
SAC: Slow adhering cells
FBS: Fetal bovine serum
PM: Proliferation medium
fMHC: Fast myosin heavy chain
DAPI: 4′, 6′ Diamidino-2-phenylindole
NBF: Neutral buffered formalin
ALP: Alkaline phosphatase
PI: Propidium iodide
FACS: Fluorescence-activated cell sorting
CMV: Cytomegalovirus
qPCR: Quantitative real-time PCR
ELISA: Enzyme-linked immunosorbent assay
3D: 3 Dimensional
Micro-CT: Microcomputed tomography
Osx: Osterix
Runx 2: Runt-related transcription factor 2
DPBS: Dulbecco’s phosphate buffered saline
Ocn: Osteocalcin
DAB: 3,3’-Diaminobenzidine
RIPA: Radioimmunoprecipitation assay buffer
PMSF: Phenylmethylsulfonyl fluoride
TBST: Tris-base buffered saline with 0.1% tween 20
ANOVA: Analysis of variance
COL1: Collagen 1
